# Dynamic facial expressions of emotions are discriminated at birth

**DOI:** 10.1371/journal.pone.0193868

**Published:** 2018-03-15

**Authors:** Margaret Addabbo, Elena Longhi, Ioana Cristina Marchis, Paolo Tagliabue, Chiara Turati

**Affiliations:** 1 Department of Psychology, University of Milan-Bicocca, Milano, Italy; 2 NeuroMi, Milan Center for Neuroscience, Milano, Italy; 3 Research Department of Clinical, Educational and Health Psychology, University College London, London, United Kingdom; 4 Neonatology and Intensive Care Unit, MBBM Foundation, San Gerardo Hospital, Monza, Italy; Universita degli Studi di Udine, ITALY

## Abstract

The ability to discriminate between different facial expressions is fundamental since the first stages of postnatal life. The aim of this study is to investigate whether 2-days-old newborns are capable to discriminate facial expressions of emotions as they naturally take place in everyday interactions, that is in motion. When two dynamic displays depicting a happy and a disgusted facial expression were simultaneously presented (i.e., visual preference paradigm), newborns did not manifest any visual preference (Experiment 1). Nonetheless, after being habituated to a happy or disgusted dynamic emotional expression (i.e., habituation paradigm), newborns successfully discriminated between the two (Experiment 2). These results indicate that at birth newborns are sensitive to dynamic faces expressing emotions.

## Introduction

We are born with a natural propensity to communicate our internal states through facial expressions: we wrinkle our nose and elevate the upper lip when we experience disgust while raising the corners of the mouth is a visible evidence of joy. Complex facial movements begin to develop already within the confines of the womb [1, 2]. By performing these facial movements the fetus provides himself with crucial motor experience for the subsequent emergence of a number of vital functions essential after birth, like breast feeding and vocalizing [3, 4]. Recent studies have also demonstrated that fetuses display facial muscle configurations that can be associated to the expression of distress and positive states and may thus be considered important components for the early interactions with the social world once the baby is born [1]. Indeed, fresh from birth newborns are capable to express internal states like distress and disgust [5], thus being able to communicate and send signals to their parents from the very first stages of their life. Closely tied to the production of facial expressions is the ability to visually discriminate between them. To date, little has been done to investigate this ability at birth.

Only a few studies have investigated newborns’ sensitivity to emotional facial expressions [6, 7, 8, 9]. Field and colleagues (1982, 1983) have shown that at birth newborns seem to be able to imitate emotional facial expressions like surprise, happiness, or sadness posed by a live model. So far, this result still remains controversial as a further attempt to replicate this finding yielded to contrasting results [10]. More recently, a couple of studies [8, 9] have explored newborns’ early ability to distinguish between different static emotional facial expressions through the visual preference and the habituation paradigms. Results from the study of Farroni and colleagues (2007) have shown that when newborns were presented simultaneously with photographs of a happy and a fearful facial expression, they preferred to look at the happy face. Conversely, newborns didn’t show any preference and didn’t even discriminate when the fearful face was compared to a neutral one. With the aim to extend these results, a subsequent study [9] has shown that when facial expressions (neutral, happy or fearful) and gaze direction (averted or directed) were combined, newborns manifested a visual preference only when happy and neutral faces with directed gaze were compared, looking longer toward the happy face [9]. The authors suggested that during the very first days of postnatal life newborns are mostly exposed to smiling faces with directed gaze and this facial expression is crucial to promote social interactions [9]. This is in line with evidence demonstrating that early in life infants are more attracted by smiling faces [11], and are facilitated in the recognition of face identity when faces display a happy emotional expression [12, 13]. However, both of these studies [8, 9] used as stimuli static photographs in which the perceptual differences between the two facial expressions were very pronounced. Therefore, it could be possible to speculate that newborns were responding to low-level perceptual features present only in the happy face (i.e. the broad toothy smile). Indeed, positive results were found only within visual preference tasks, which are particularly affected by the presence of salient perceptual features early in life [14]. Also Kestenbaum and Nelson (1990) showed that when a salient feature like a toothy smile was available, discriminative abilities of emotional facial expressions were driven by this perceptual attribute even at 7 months of age [15].

Most importantly, much of the current knowledge about visual processing of facial expressions in infancy as well as in adulthood comes from studies that have used static stimuli. But, during our daily interactions, especially in our first days of life, we mainly encounter facial expressions that unfold over time. In the last decades many researchers started to comprehend the importance of studying facial expressions in the way they naturally take place in everyday life (i.e. dynamic) [16] and this shift of attention from static to dynamic stimuli led to a number of relevant findings. First of all, there is evidence that static and moving faces are processed differently: both adults [17] and infants [18, 19, 20] scan dynamic and static faces in a different way. Further, dynamic displays of facial expressions activate in adults different brain areas compared to still pictures [21, 22]. Interestingly, dynamic displays improve adults’ accuracy in emotion recognition tasks especially when visual information is limited and degraded [23, 24]. This finding suggests that also newborns, whose visual system in the first few days of life is very immature and characterized by a poor spatial resolution and contrast sensitivity [25], could benefit from the information coming from facial dynamics when perceiving emotional expressions. Facilitative effects of motion related information can be found already at birth. For example, newborns can perceive illusory contours [26] and discriminate between possible and impossible hand movements [27] only when dynamic information is available. In addition, Bulf and Turati (2010) have shown that newborns successfully use information coming from rigid head movements to recognize a face identity posed in a novel viewing perspective [28]. In line with these results, Leo and colleagues (2017) have also recently found facilitatory effects of semi-rigid movement associated with facial expressions in identity recognition at birth [29]. In contrast, a couple of findings with newborns have shown that face movements alone (i.e. talking) don’t favour face recognition [30, 31]. These contrasting results could be explained by the nature of the different type of stimuli employed (e.g. rigid head movements as in Turati et al., 2010, movements associated to emotional expressions as in Leo et al., 2017 versus talking motions as in Coulon et al., 2011 and Guellai et al.2011) which could have different influences on face recognition.

To sum up, so far there’s little evidence concerning newborns’ ability to discriminate others’ emotional expressions. Moreover, the few studies that have directly explored this ability in newborns have used exclusively static face pictures [8, 9]. At birth, newborns possess relatively little visual experience and what they actually see in their first days of life are dynamic facial expression and not static faces. To date, it still remains unknown whether at birth newborns are sensitive to others’ dynamic facial movements that express emotions. When we express an emotion, a combination of internal features of our face (i.e. eyes, nose, cheeks, mouth) dynamically change configuration over time. Each emotion is the result of a particular combination of these internal features. Are newborn infants able to detect the morphological changes that take place during the unfolding of a facial expression? Are they able to discriminate between different emotional dynamic expressions and do they already possess spontaneous visual preferences for a dynamic facial expression over the others?

Here we explore whether 2-day-old newborns are sensitive to facial movements expressing happiness and disgust using a visual preference (Experiment 1) and a habituation task (Experiment 2) to test respectively newborns’ spontaneous visual preferences and discriminative abilities. The happy and disgusted expressions are conveyed by information coming from a combination of movements in different facial regions (i.e. eyes, mouth) which are comparable in saliency (i.e. narrowing of the eyes, opening of the mouth). As a result, in order to discriminate between these facial emotional expressions, newborns cannot rely on highly salient facial features (i.e. eyes wide open, visible teeth), as they considerably and dynamically vary in both the happy and the disgusted facial expression. Rather, newborns’ ability to discriminate between the happy and disgusted dynamic facial expression can only rely on the information coming from the face movements related to the two emotional facial expressions.

## Experiment 1

In this first study we used a visual preference task to explore whether newborns can manifest a visual preference when presented simultaneously with two different dynamic displays depicting a smiling face and a disgusted one. We expect them to show a visual preference for the smiling face, as shown by previous studies with newborn and older infants [8, 9, 32].

### Participants

Eighteen healthy full-term Caucasian newborns (9 girls; mean age: 44 h, range: 21–83, mean birth weight: 3328 g, Apgar score: at least 8 after 5 minutes from birth) recruited at the maternal unit of the S. Gerardo Hospital of Monza were tested when they were in an awake and alert state. We have tested additional 4 newborns but they were then excluded from the final sample due to fussiness or not being cooperative (n = 3) or to a position biased (i.e. looking towards the right or the left position for more than the 85% of the total looking time) (n = 1). Parental written informed consent was obtained before testing began. The protocol was carried out in accordance with the ethical standards of the Declaration of Helsinki (BMJ 1991; 302: 1194) and approved by the Ethics Committee of the S. Gerardo Hospital of Monza (NeoViper, n.1531/2011).

### Stimuli

**Fig 1:**
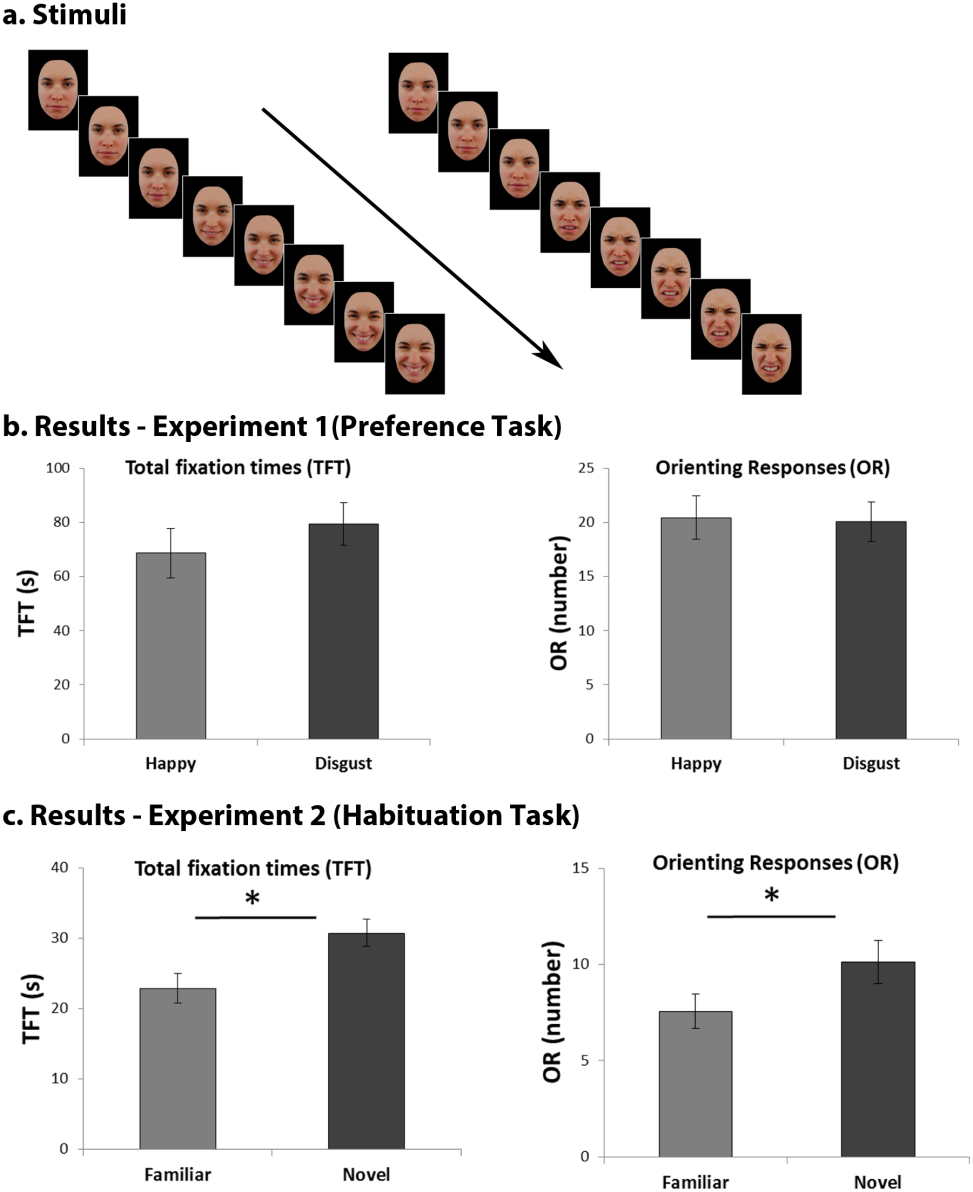
Stimuli and results. **a)** Frames composing the videos depicting the expression of disgust (left) and happiness (right) of one of the two face identities shown. Frames are presented in succession in the direction indicated by the arrow. The actress in the photograph has given written informed consent to publication of her photograph. **b)** Newborns’ total looking times (left) and orienting responses (right) towards the expression of happiness and disgust in Experiment 1 (Preference task). **c)** Newborns’ total looking times (left) and orienting responses (right) towards the novel and the familiar expression in the test phase of Experiment 2 (Habituation task). Error bars refer to the standard errors of the mean. * = p<.05.

Newborns were presented simultaneously with two color videos of a woman’s face performing a happy and a disgusted expression on a black background. The faces of two different Caucasian women were used (face A and face B). Two face identities were employed as stimuli in order to avoid the possibility that newborns’ visual preference could be ascribed to the salient features of one single identity expressing an emotional expression. Half of the newborns saw face A and the other half saw face B, randomly assigned. In both identities women had a direct gaze and their hair, ears and neck as well, were not visible. Each video lasted 4568 ms and was made of 8 frames, each one of the duration of 571 ms. The first two frames depicted the face with a neutral expression and then, in the following 6 frames, the happy/disgusted expression unfolded and reached the maximum intensity in frame 8 (Fig 1). The two videos were shown bilaterally at a distance of 27°, and played continuously, in a loop. At a distance of 30 cm from the screen face A was 24° wide and 33° high, and face B was 24.8° wide and 33° high. The diameter of the iris was 1.9° for both faces A and B. The Luminance, contrast, and hue, as well as saturation, were kept constant between the frames and the stimuli.

Stimuli were validated by 32 adults (16 females, mean age = 23.7, SD = 4.06) who were asked to rate the emotional valence of the stimuli on a 10-point Likert scale, where -5 and +5 indicate maximum disgust and maximum happiness respectively. Results showed that the happy face was judged as expressing happiness in both identities A (M = 3.50, SD = 1.72) and B (M = 4.03, SD = 0.93). Also the disgusted face was judged to express disgust in both faces A (M = -3.91, SD = 1.76) and B (M = -3.84, SD = 0.88). The Wilcoxon test indicated that there was a significant difference between the judgements of the emotional valence of the two stimuli (Face A, p < .001 and Face B, p < .001). Further, no significant difference was found between the ratings of the two happy faces (happy face A and happy face B, Wilcoxon test, p > .19) and between the two disgusted faces (disgusted face A and disgusted face B, Wilcoxon test, p > .33).

### Procedure

Newborns were seated on the lap of an undergraduate student unaware of the aims of the study, at a distance of 30 cm from the stimulus presentation monitor (27” screen size, 1920 X 1080 pixel resolution, 60 Hz). A camera was placed above the monitor that recorded newborn’s gaze thus allowing an experimenter to code online newborns’ visual behavior. The baby holder could check if newborns’ position was aligned to the center of the screen on a monitor that displayed his/her face. Total looking times and number of orienting responses were measured within a preferential looking paradigm with an infant-control procedure [8, 9]. Newborns were presented with two trials, in which a happy and a disgusted face were displayed simultaneously and bilaterally on the screen. Each trial began as soon as the newborns looked at a red flickering circle appearing in the center of the monitor. The left/right position of the stimuli was reversed between the first and the second trial and in the first trial half of the newborns saw the happy face on their right and the other half on their left. Each trial ended when the newborns watched each stimulus at least once, and shifted their gaze away for more than 10 s. Half of the video-recordings of eye movements were coded offline by an observer, blind to the stimuli shown. The inter-coder agreement (Pearson correlation) was .97 for total fixation time and .82 for number of orientations. The Intra-Class Correlation (ICC) coefficient was .98 for total fixation time and .85 for number of orientations, revealing an excellent absolute agreement between coders.

### Results

A repeated-measures Analysis of Variance (_rm_ANOVAs) was performed with *trial* (first vs. second) and *emotion* (happy vs. disgust) as within-subjects factors. The analysis showed no significant main effects or interaction both in total looking times (all ps > .295) and eye orienting responses (all ps > .187) (Fig 1). Thus, contrary to our hypothesis, results of Experiment 1 show that newborns didn’t prefer a facial expression over the other, as there was no significant difference between the amount of time they have spent looking towards the face expressing happiness (M = 68.6 s; SD = 37.3) and disgust (M = 79.4 s; SD = 33.7). The number of fixations towards the happy (M = 20.4; SD = 8.6) and the disgusted face (M = 20.1; SD = 7.7) did not significantly differ as well.

## Experiment 2

The lack of preference shown by newborns in Experiment 1 doesn’t directly imply that they weren’t able to differentiate between happy and disgusted faces. It is possible that newborns simply didn’t prefer an emotion over the other, even if they were able to discriminate between them. To ensure whether newborns were capable to distinguish between the two dynamic expressions despite the null result revealed during the preference task, we conducted a second experiment in which we used a visual habituation paradigm. We habituated newborns to happy or disgusted facial expressions. Following the habituation phase, we tested their discriminative abilities presenting them the familiar expression along with the novel one. If newborns are able to discriminate between the two facial expressions, then we expect them to look longer and orient their gaze more frequently towards the novel stimulus in the test phase, regardless of the facial expression presented in the habituation phase.

### Participants

Eighteen healthy full-term newborns recruited at the maternal unit of the S. Gerardo Hospital of Monza (11 girls; mean age: 46 h, range: 24–82, mean birth weight: 3349g, Apgar score: at least 8 after 5 minutes from birth) were tested when they were in an awake and alert state. We have tested other 6 newborns but they were then excluded from the final sample due to fussiness or not being cooperative (n = 4) or to a position biased (i.e. looking towards the right or the left position for more than the 85% of the total looking time) (n = 2). Parents signed a written informed consent before testing began. The protocol was carried out in accordance with the ethical standards of the Declaration of Helsinki (BMJ 1991; 302: 1194) and approved by the Ethics Committee of the S. Gerardo Hospital of Monza (NeoViper, n.1531/2011).

### Stimuli

Stimuli were the same as in Experiment 1.

### Procedure

The experimental setting was the same as in Experiment 1. Newborns were tested using a visual habituation paradigm and total looking times and orienting responses were measured [14]. The habituation phase started when the newborn oriented towards a red flickering circle appearing in the center of the screen. During the habituation phase, newborns viewed bilaterally two videos of a face expressing the same emotion (happy or disgust). Half of the newborns were habituated to the happy faces, the other half to the disgusted faces. As in Experiment 1, two identities were used (face A and face B). In each habituation trial, videos were shown continuously in a loop until newborns shifted their gaze away for more than 2 s. Habituation phase ended when the newborn reached the habituation criterion which was a 50% decline in looking time on the last three consecutive trials, relative to the looking time on the first three trials. Following habituation, newborns were presented with two test trials in which a novel facial expression (happy for newborns habituated to disgusted facial expressions and vice-versa) and a familiar facial expression were displayed simultaneously and bilaterally on the screen. Each test trial began as soon as the newborns looked at a red flickering circle appearing in the center of the monitor. The left/right position of the stimuli was reversed between the first and the second trial and in the first trial half of the newborns saw the happy face on their right and the other half on their left. Each test trial continued in a loop until newborns looked for a minimum of 20 s, each stimulus was watched at least once and newborns’ gaze was shifted away for more than 500 ms. This procedure has previously been used in several studies with newborn infants [28, 26]. Half of the video-recordings of eye movements were coded offline by an observer, blind to the stimuli shown. Inter-coder agreement (Pearson correlation) was .91 for total fixation time and .84 for number of orientations. The Intra-Class Correlation (ICC) coefficient was .96 for total fixation time and .87 for number of orientations.

### Results

All newborns reached the habituation criterion. In fact, a repeated-measures Analyses of Variance (_rm_ANOVAs) on total looking times with *habituation condition* (happy vs. disgust) as the between-subjects factor, and *habituation trials* (first three vs. last three) as the within-subjects factor revealed a significant effect of habituation trials, *F*(1,16) = 31.3, *p* < .001, η_p_^2^ = .662. The average looking time on the first three habituation trials (*M* = 54.8 s, SD = 29.7) was significantly longer than the average looking time on the last three habituation trials (*M* = 17.2 s, SD = 9.9). No other effect was significant. On average, newborns required 6.75 trials to habituate to the happy face, and 7 trials to habituate to the face expressing disgust.

A repeated-measures Analyses of Variance (rmANOVAs) on total looking times with *trial* (first vs. second) and *novelty* (novel vs. familiar) as within-subjects factors and *habituation condition* (happy vs. disgust) as between-subjects factor, revealed a main effect of novelty, *F*(1,16) = 4.87, *p* = .042, η_p_^2^ = .233. Infants looked significantly longer at the novel (*M* = 30.7 s, *SD* = 8.5) than the familiar (*M* = 22.8 s, *SD* = 9.2) facial expression during test phase (Fig 1). The other factors and interactions were not significant (all ps > .154). This result was further confirmed by examination of the data for individual infants, showing that 13 of the 18 infants in the sample looked longer at the novel stimulus than at the familiar one (binomial test, p = .033).

A repeated-measures Analyses of Variance (rmANOVAs) on eye orienting responses with *trial* (first vs. second) and *novelty* (novel vs. familiar) as within-subjects factors and *habituation condition* (happy vs. disgust) as between-subjects factor revealed a main effect of novelty, *F*(1,16) = 7.31, *p* = .016, η_p_^2^ = .314. Infants oriented more frequently towards the novel (*M* = 10.1 s, *SD* = 4.7) than towards the familiar (*M* = 7.5 s, *SD* = 3.7) facial expression during test phase (Fig 1). No other significant effect or interaction emerged from this analysis (All ps > .071). These results show that newborns are able to discriminate between a dynamic happy and disgusted expression, as indicated by longer looking times and more frequent eye orientations towards the novel stimulus in the test phase.

## Discussion

In the present study we have tackled the intriguing question of whether in the first stages of postnatal life newborns are able to discriminate between dynamic displays of emotional facial expressions. This is the first study to address the issue of newborns’ ability to visually discriminate emotional expressions using dynamic face stimuli. We have found that when 2-day-old newborns were presented simultaneously with moving faces expressing happiness and disgust, they didn’t manifest a spontaneous visual preference toward one of the two stimuli (Experiment 1). We have also demonstrated that the absence of a preference response in Experiment 1 wasn’t explained by a general inability at birth to discriminate between the two dynamic emotional expressions. Newborns were actually able to differentiate between a happy and a disgusted moving face as testified by an overall preference in looking times and orienting responses towards the novel facial expression in the visual habituation task (Experiment 2).

Young infants typically prefer to look at happy faces at least until they reach the age in which they start to locomote (i.e. 7 months of age) and this interest for happy faces has been interpreted as the result of infants’ early experience with smiling faces [11]. Preferences for happy faces were also found at birth, at least in some limited circumstances [8, 9]. However, it is possible that, in these studies [8, 9], newborns were responding to a very salient feature like the toothy smile. Here we have shown that newborns didn’t manifest any preference when highly salient facial features were controlled and when emotions were presented in a dynamic fashion. How can we explain newborns’ lack of preference found in Experiment 1? One possible explanation can be related to the fact that dynamic displays represent extremely salient stimuli for newborns and the effect of kinematics on their discriminative abilities could be beneficial but also distracting. For example, recent findings [30, 31] suggest that the dynamics of talking faces were so attractive for newborn infants to the point of interfering with their ability to process properly the differences between face identities. Accordingly, emotional facial dynamics in the present study might have been too engaging to reveal a visual preference response towards one of the two emotional expressions. However, this interpretation needs to be further confirmed by directly comparing static and dynamic facial expressions. So far studies with newborns seem to converge in considering motion as a cue that facilitates rather than interfere with newborns’ perceptual abilities [26–29]. Thus, an alternative and more plausible interpretation of our results is that newborns were actually able to distinguish between the two stimuli during the preference task but they simply did not prefer a dynamic facial expression over the other. Both emotional dynamics might have attracted equally their attention. It could be speculated that, when stimuli are well controlled, as in our study, newborns do not show to possess spontaneous preferences for happy expressions over the others. This supports the idea that preferences for happy faces found in previous studies with newborns [8, 9] were more likely driven by highly salient facial features.

Experiment 2 has confirmed that newborns were able to discriminate between two different dynamic faces expressing happiness and disgust. This indicates that 2-day-old newborns are already able to detect the changes that take place during the unfolding of an emotional facial expression. This is in line with several studies showing that the visual system is already sensitive to motion-based information at birth [26, 27, 28].

One could speculate that the different results found in Exp. 1 and 2 (lack of preference in Experiment 1 and successful discrimination in Experiment 2) may be explained by methodological differences, such as the duration of the test trials, which is longer in Experiment 1 and shorter in Experiment 2. However, this possibility doesn’t seem tenable for two different reasons. First, it is well known that infant control procedures maximize rather than minimize newborns’ ability to manifest a visual preference [14, 33]. Secondly, in Experiment 1 newborns don’t manifest any preference (all ps > .23) even when we analyze total looking times using the same criteria of Experiment 2 (cumulative looking time of 20 sec, each stimulus looked at at least once and 500 ms look-away).

Overall, our results imply that newborns’ discriminative abilities are sophisticated enough to allow them to distinguish between two different, complex dynamic facial expressions. When Farroni and colleagues (2007) compared static facial expressions where perceptual differences weren’t very marked (i.e. fearful vs. neutral faces), newborn failed to show a preference and even to discriminate between them [8]. It’s reasonable to think that small differences between static facial expressions might become undetectable by newborns’ very immature visual system, which might benefit by the presence of perceptual information conveyed by dynamic emotional faces. However, our results don’t allow us to draw firm conclusions about the facilitating influence of dynamics on emotion discrimination and future studies should address this issue by directly comparing newborns’ ability to discriminate dynamic and static stimuli.

The ability to discriminate facial expressions develops very early in human life [11, 34] and is considered a prerequisite for emotion recognition, which matures later in development. For instance, only by 7 months of age infants seem capable to truly attribute an affective meaning to emotional expressions [35, 36]. Then, only at the end of the first year of life develops the ability to use the information coming from their caregiver’s facial expression to understand what is safe or harmful in the environment, and act accordingly (i.e., social referencing)[37]. Our results show that, despite the relatively little visual experience that newborns possess at birth, they are already able to distinguish between different dynamic facial expressions and this early ability might provide a fertile ground for the development of later and more complex cognitive skills. However, visual experiences accumulated in the first days of life may not be enough to boost newborns’ preference towards one of the two dynamic emotional expressions. Significant postnatal experiences may be required to refine and enrich infants’ processing of dynamic emotional expressions.

Investigating how newborns process dynamic emotional facial expressions is fundamental to fully understand how they actually see and process the social world around them, which is constantly in motion. Our results show that at birth infants are able to distinguish between different emotional facial dynamics and, thus, this study represents a crucial step towards a deeper comprehension of newborns’ sensitivity to human emotional facial behavior.
